# Energy response of EBT3 radiochromic films: implications for dosimetry in kilovoltage range

**DOI:** 10.1120/jacmp.v15i1.4439

**Published:** 2014-01-06

**Authors:** J. Eduardo Villarreal‐Barajas, Rao F.H. Khan

**Affiliations:** ^1^ Department of Oncology and Department of Physics and Astronomy University of Calgary , Calgary AB; ^2^ Department of Medical Physics Tom Baker Cancer Centre Alberta Canada

**Keywords:** radiochromic film dosimetry, EBT3 films, kilovoltage dosimetry, energy response, orthovoltage dosimetry

## Abstract

The objective of this study is to evaluate the suitability of recently introduced radiochromic film EBT3 for clinical dosimetry in the kilovoltage (kV) range. For this purpose, a kV X‐ray irradiator, X RAD 320ix in the range 70 to 300 kVp, a clinical ${}^{60}\text{Co}$ source, a 6 MV and an 18 MV X‐ray clinical beam from a Varian linear accelerator were calibrated following AAPM dosimetry protocols. EBT3 films from two different EBT3 batches were placed side‐by‐side on the surface of a water phantom; doses from 0.5 to 4 Gy were delivered. Similarly, irradiations were performed for ${}^{60}\text{Co}$ and 6 and 18 MV beams in a water‐equivalent phantom. Films were reproducibly placed at the center of a flatbed scanner and 48‐bit RGB scans were obtained both pre‐ and postirradiations. Net optical density (netOD) and response for a given radiation quality relative to ${}^{60}\text{Co}$ was determined for each EBT3 film. The netOD of the red color showed reproducible response (within 1%) for both batches when irradiated using the ${}^{60}\text{Co}$ source. For a given dose of 1 Gy of kVp X‐ray, the response relative to ${}^{60}\text{Co}$ using the three color channels (red, green, and blue) decreases with decrease in kVp, reaching a maximum underresponse of ∼20% for the 70 kVp. A significant underresponse of ∼5% was observed at 300 kVp. Responses of MV X‐ray beams with respect to ${}^{60}\text{Co}$ at the 1 Gy dose level showed no statistically significant difference. A relatively small difference in the response was observed between the two EBT3 batches used in this study in the kV X‐ray range.

PACS numbers: 87.56.B, 87.57.uq

## I. INTRODUCTION

EBT3 is the most recently released radiochromic film by International Specialty Products Ashland Inc. (Covington, KY) for applications in clinical dosimetry. According to the manufacturer, EBT3 compared to its predecessor EBT2 presents two major improvements: film symmetry and an anti‐Newton ring coating. Similar to EBT2 and EBT, EBT3 exhibits highest sensitivity (higher absorbance) at 636 nm; therefore, if the film is scanned for dose evaluation, the maximum sensitivity is obtained by using the red channel. According to the manufacturer, the red channel is recommended for dose evaluations up to 8 Gy, while the green channel can be used for doses from 8 to 40 Gy. The blue channel provides a response signal to automatically correct for the nonuniformity of the film by incorporating a special marker dye in the active layer of the EBT3 films. EBT3 films have a 30‐micron active substrate layer sandwiched between two matte polyester layers of 125 microns each. The sensitive emulsion of the new EBT3 film has the same composition^(1)^ as its predecessor EBT2.^(2)^

There have been only two publications addressing the energy response of the EBT3 films in the kilovoltage (kV) range.^3^, ^4^ The energy dependence results reported in these investigations indicate a small to negligible energy dependence for EBT3 films when exposed to monoenergetic and polyenergetic kV X‐ray beams. The aim of the current work is to systematically evaluate energy dependence of EBT3 films in terms of the ratio of net optical density (netOD) of the X‐ray beam with respect to the netOD of ${}^{60}\text{Co}$, and determine the EBT3 dose response for clinically relevant orthovoltage X‐ray beams of varying beam quality (70‐300 kVp). The choice of ${}^{60}\text{Co}$ as a reference was made due to its reliable absolute dosimetry. Given the significant variation in the response of different EBT and EBT2 batches previously observed by Lindsay et al.^(5)^ and the energy dependence response findings from the Monte Carlo based study of the same films by Sutherland and Rogers,^(2)^ it was decided to use two EBT batches for this investigation in order to verify their consistency.

## II. MATERIALS AND METHODS

### A. Irradiation setup and dosimetry

**Figure 1:**
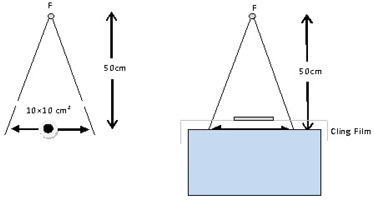
kV dosimetric calibration and corresponding radiochromic film irradiation setups where F is the focus of the X‐ray source.

All EBT3 film irradiations for the kVp beams were performed on a research X‐RAD 320ix X‐ray irradiator (Precision X‐Ray Inc, North Brantford, CT). The quality (HVL) of the kVp beams was determined using narrow beam geometry, as recommended by the TG61 report.^(6)^ Table 1 list the main characteristics of the kVp beams used in this investigation. The X‐RAD 320ix irradiator was calibrated using an in‐air technique as described in the AAPM TG61 report. The absorbed dose to water at the surface was measured at focus‐to‐skin distance (FSD) of 50 cm (defined at the center of the chamber) for a field size of $10\times 10\;{\text{cm}}^{2}$ with an Exradin A19 (Standard Imaging, Middleton, WI) ionization chamber (Fig. 1). The Exradin A19 ion chamber was calibrated at the National Research Council of Canada (NRCC). The NRCC calibration certificate included beam qualities ranging from HVL=1.5 mm Al to HVL=1.5 mm Cu (60 to 250 kVp, respectively). $N_{K}$ values required for the absorbed dose to water at the surface, for each kVp beam, were obtained by interpolation of the NRCC $N_{K}$ values included in the calibration certificate. The $N_{K}$ values ranged from 45.8 mGy/nC to 44.8 mGy/nC for the lowest to the highest kVp beams. The Exradin A19 chamber exhibited $P_{\text{ion}}$ and $P_{\text{pol}}$ values ranging from .997 to 1.003 for all the kVp beams under investigation. The water kerma‐based backscatter factor and the ratio of water to air average mass energy absorption coefficients were obtained from the TG61 report.^(6)^

The EBT3 film irradiations were performed in the same geometry as described for the assessment of the dose to water at the phantom surface using the ionization chamber, with the exception that the films were placed at FSD = 50 cm on the surface of a $15\times 15\times 15\;{\text{cm}}^{3}$ water tank. The EBT3 films were supported on the water surface in the center of a $10\times 10\;{\text{cm}}^{2}$ field by ∼15 micron thick cling plastic film (Fig. 1). The absorbed dose to water at the surface was determined from the films without correcting for the attenuation caused by the 125 micron polyester top layer of the EBT3 film or for the influence on the backscatter produced by the 15 micron cling film used for the EBT3 film irradiation setup. The maximum attenuation from the polyester top layer of EBT3 film approximates to 0.7% for the lowest X‐ray energy used in this study (70 kVp, effective energy 32 keV).

**Table 1:** Radiation beam characteristics employed in experiments. Effective energies were derived using the measured beam qualities and the mass attenuation coefficient tables published by NIST.^(11)^

*E (kVp)*	*Added Filtration*	*Beam Quality (HVL)*	*Effective E (keV)*	*Dose Rate (Gy/min)*
70	2 mm Al	3.0 mm Al	32	0.316
100	2 mm Al	4.2 mm Al	38	0.682
150	2 mm Al	6.3 mm Al	47	1.538
200	0.75 mm Sn +0.25 mm Cu+1.5 mm Al	2.6 mm Cu	132	0.392
300	0.75 mm Sn+0.25 mm Cu+1.5 mm Al	4.0 mm Cu	168	0.824
Co‐60			1250	1.250

The EBT3 film irradiations were also performed on a clinical ${}^{60}\text{Co}$ unit (Theratron 780‐C; Best Theratronics, Ottawa, ON). The ${}^{60}\text{Co}$ dose rate and the irradiation time were determined by performing a dose calibration following the AAPM TG51 protocol guidelines.^(7)^ The absorbed dose to water on this ${}^{60}\text{Co}$ unit has also been previously verified using the TLD verification services offered by the Radiation Dosimetry Services (Houston, TX). The ${}^{60}\text{Co}$ EBT3 film irradiations were performed isocentrically in a solid water (Gammex Middleton, WI) phantom at a depth of 10 cm for a $10\times 10\;{\text{cm}}^{2}$ field. To provide adequate backscatter for the ${}^{60}\text{Co}$ irradiations, another 10 cm of solid water slabs were placed behind the EBT3 films. Additional 6 and 18 MV X‐ray irradiations were performed using the same setup as the one described for the ${}^{60}\text{Co}$ irradiations (except that the source‐to‐axis distance, SAD, was set to 100 cm instead of the 80 cm used for the ${}^{60}\text{Co}$ irradiations). The solid water slabs used for all the ${}^{60}\text{Co}$, 6, and 18 MV irradiations had dimension of $30\times 30\;{\text{cm}}^{2}$ and 5 cm thickness.

Our studies included two radiochromic EBT3 film batches: referred as B1 (Lot #A12141101, expiration date December 2013) and B2 (Lot #A05151203, expiration date May 2014). Identical irradiations were done with ${}^{60}\text{Co}$ and kVp X‐ray beams at doses ranging from 0.5 Gy to 4 Gy for both batches B1 and B2. All irradiations were repeated twice, resulting in two independent irradiations and, therefore, two netOD evaluations for every beam energy and batch. The irradiation were performed by placing pieces of films ($\sim 4\times 4\;{\text{cm}}^{2}$) on top of each other, setting B1 films on top in the first irradiation and B2 films on top for the repeated (second) irradiation. Prior to each kVp X‐ray irradiation, the dose to water at the surface was confirmed using A19 Exradin ionization chamber in order to assess the output stability of the X‐ray unit at the time of irradiation. The largest X‐ray output variation observed during the course of all of our experiments was 1.2%.

### B. Film processing and image analysis

The netOD for all the films was obtained from the scanned images using an EPSON 10000XL flatbed scanner (US Epson, Long Beach, CA). The films were scanned with transmission mode (positive film mode), 48 bits RGB (16 bits per channel color), 72 dpi resolution (0.35 mm/pixel), without any image correction. All of the films were approximately $4\times 4\;{\text{cm}}^{2}$. The films were marked to maintain the same orientation at the time of scanning. A template was used to position the films at the center of the scanner in a reproducible manner. The template was removed during the scanning. The scans were performed approximately 24±4 hours after the irradiations. Both irradiations and scanning were done at room temperature ($21^{\circ }C\pm 2^{\circ }C$). The films were stored before and after irradiations in opaque envelopes inside a dark lined cardboard box to reduce exposure to light. Freely available image processing and analysis software, ImageJ (http://rsbweb.nih.gov/ij/) was used to extract the netOD by obtaining the average pixel number from a $1\times 1\;{\text{cm}}^{2}$ region of interest (ROI) around the center of each $4\times 4\;{\text{cm}}^{2}$ film. All films were scanned prior to any irradiation in order to extract the zero dose (unexposed) intensity. The netOD for the red, green, and blue channels (${\text{netOD}}_{R},\;{\text{netOD}}_{G},\;{\text{netOD}}_{B}$) was derived from the average pixel numbers following Devic et al.^(8)^ However, only the ${\text{netOD}}_{R}$ was used for dose evaluations. Calibration curves for all beam energies were obtained by plotting the ${\text{netOD}}_{R}$ vs. dose (cGy). The uncertainty in the netOD was determined by the standard deviation of the mean pixel value obtained from the ROI for each color.

In order to quantify the EBT3 energy dependence, the ratio of the netOD (Rx) associated with a given dose of the X‐ray beam (${\text{netOD}}_{\text{kVp}}$) under investigation relative to the netOD for the same dose for ${}^{60}\text{Co}$ (${\text{netOD}}_{\text{Co}-60}$) was used as a metric:

(1) $$\text{Rx}=\frac{\text{netOD}(D,\text{xkVp})}{\text{netOD}(D,\text{Co}-60)}$$

This will henceforth be referred to as the response ratio. The choice of clinical ${}^{60}\text{Co}$ source as a reference was made due to well‐known radioactive decay characteristics, stable dose rate, and availability at our cancer center.

As an independent check of the energy response of the films, dose estimates for the kVp irradiated films were obtained using a ${}^{60}\text{Co}$ calibration curve. These ${}^{60}\text{Co}-\text{based}$ dose estimates provide a direct indication of the energy dependence of the EBT3 films in terms of the error introduced in the dose assessment of kVp irradiated films if there was no energy dependence with respect to ${}^{60}\text{Co}$. The term dose in the present work is referred to as absorbed dose to water, unless otherwise stated.

## III. RESULTS

The ${}^{60}\text{Co}$ exposed films from both batches (B1 and B2) showed consistently the same response (within 1%) in terms of the ${\text{netOD}}_{R}$, as shown in Table 2. However, the ${\text{netOD}}_{G}$ and ${\text{netOD}}_{B}$ were on average 2.5% and 5.9% higher for the B2 with respect to B1 for ${}^{60}\text{Co}$ irradiation. Similarly, Table 3 shows the ratio of netOD from B2 to B1 resulting from 1 Gy of irradiation with kVp beams. Overall the differences in the ${\text{netOD}}_{R}$ between the two EBT3 batches decreases as the kVp energy increases and reaches a minimum difference of 2% for 300 kVp beam. However, the green channel showed an almost constant B2/B1 ratio of 1.06, regardless of the kVp. The blue channel exhibited no clear trend on the B2/B1 ratio. In general, we observed a systematically higher ${\text{netOD}}_{R}$ and ${\text{netOD}}_{G}$ for B2 compared to B1 for all the investigated kVp X‐ray beams. The reproducibility in terms of average pixel number on the $1\times 1\;{\text{cm}}^{2}$ scored area for the two independent irradiations performed for every kVp irradiation were within 0.5% for the three color channels.

The response ratios, Rxs, for the three colors for all the kVp X‐ray qualities investigated at 1 Gy of absorbed dose are shown in Table 4. From these results it is evident that the Rx for the red color monotonically decreases from 300 kVp to the 70 kVp. Rx reaches a maximum of 0.95 for B2 at 300 kVp and a minimum of 0.79 for B1 at 70 kVp. An examination of the Rx for the red channel from Table 4 also shows a consistently higher value for B2 compared to B1; however, these differences are significantly reduced for 300 kVp. The Rx‐green and Rx‐blue in general have B2 higher than B1. The increasing Rx trend with kVp holds for the Rx‐green and the Rx‐blue.

**Table 2:** Ratio of net optical densities for two batches of EBT3 for ${}^{60}\text{Co}$ irradiation

	$netOD_{B2}/netOD_{B1}$		
*Dose (cGy)*	*Red*	*Green*	*Blue*
50	0.99±0.02	1.03±0.04	1.10±0.07
75	1.00±0.01	1.03±0.03	1.02±0.06
100	1.00±0.01	1.02±0.02	1.09±0.06
200	1.00±0.01	1.03±0.02	1.07±0.05
400	0.99±0.01	1.02±0.01	1.02±0.04
Mean	0.997	1.025	1.059

**Table 3:** Ratio of netOD for two batches of EBT3 film for kVp energies for 1 Gy of absorbed dose

	$netOD_{B2}/netOD_{B1}$		
*kVp*	*Red*	*Green*	*Blue*
300	1.02±0.04	1.06±0.06	1.15±0.19
200	1.03±0.04	1.06±0.05	1.15±0.16
150	1.05±0.03	1.05±0.06	1.06±0.13
100	1.05±0.04	1.06±0.06	1.08±0.15
70	1.06±0.04	1.06±0.05	1.06±0.16
Mean	1.04	1.06	1.10

**Table 4:** Response ratio, Rx, of various kVp beams relative to ${}^{60}\text{Co}$ for B1 and B2 batches for 1 Gy of absorbed dose

	*B1(B2): Rx*		
*kVp*	*Red*	*Green*	*Blue*
300	0.94±0.03(0.95±0.03)	0.93±0.05(0.96±0.04)	0.83±0.13(0.89±0.14)
200	0.91±0.02(0.93±0.03)	0.90±0.04(0.93±0.03)	0.82±0.11(0.86±0.12)
150	0.83±0.02(0.87±0.02)	0.83±0.04(0.85±0.04)	0.75±0.09(0.74±0.10)
100	0.83±0.03(0.86±0.03)	0.83±0.04(0.85±0.04)	0.76±0.10(0.75±0.11)
70	0.79±0.03(0.83±0.02)	0.80±0.04(0.82±0.03)	0.74±0.12(0.72±0.10)

**Figure 2:**
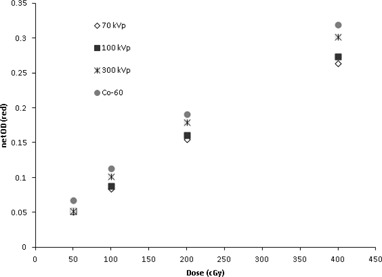
Variation of netOD with dose (red channel only) for EBT3 radiochromic films. The error bars for all points are smaller than the markers.

Figure 2 presents the results of the ${\text{netOD}}_{R}$ versus dose (EBT3 film calibration) for 70, 100, and 300 kVp and for ${}^{60}\text{Co}$ for doses in the 0.5 to 4 Gy range. It is worth noting that the Rx increases with increasing dose for all kVp beams. This Rx increase with dose is similar to that observed by Lindsay et al.^(5)^ on EBT film irradiated at 105 and 220 kVp X‐ray energies. The minimum change in Rx with increasing dose is observed for the 70 kVp beam (from 0.76 at 0.5 Gy to 0.83 at 4 Gy), while the maximum increase was observed for the hardest beam, 300 kVp (from 0.78 at 0.5 Gy to 0.94 at 4 Gy).

## IV. DISCUSSION

The Rx results for the EBT3 batches investigated in the present study demonstrate significant energy dependence for this new radiochromic film. The error in terms of the kVp dose predicted using a ${}^{60}\text{Co}$ calibration for the red channel can result in up to 22% underestimation of the delivered dose for the lowest beam quality studied in this work, 70 kVp at a dose level of 1 Gy. The underestimation is relatively smaller for 300 kVp beam, accounting for an approximately 5% for the two EBT3 batches studied. The systematic but statistically insignificant difference in Rx observed for EBT3 batches may be caused by differences in the thickness of sensitive emulsion between batches or by a small difference in the batch‐to‐batch sensitive emulsion composition. This observation is supported by the fact that the Rxs tend to diverge at the lower kVp while converging at the highest beam energies. The Rx deviation between the two EBT3 batches is, however, relatively smaller than reported by Lindsay et al.^(5)^ for EBT and EBT2 films. This relatively small difference suggests that a per batch film calibration may be required in order to achieve the desired accuracy for clinical dosimetry of kVp X‐ray beams.

The observed EBT3 underresponse, particularly noticeable below 100 kV X‐ray beams, can be attributed to the differences in material composition of the sensitive emulsion with respect to water. The small but systematic difference in the response of the two studied EBT3 batches may be explained by differences in their sensitive emulsion composition or sensitive emulsion thickness.^(2)^ The emulsion composition differences can also change the intrinsic detector response, which may contribute to the observed difference in the response between the two batches used in this investigation.

The preirradiation optical densities (OD) derived from the blue channel were consistently 0.381±0.002. This consistency can be interpreted as a consistency in the thickness of the sensitive emulsion between the studied batches.^(9)^ A recent study by Brown et al.^(3)^ suggested that, for monochromatic X‐rays of 25 kV, 30 kV, and 35 kV, the relative sensitivity (defined as ${\text{netOD}}_{\text{xkV}}/{\text{netOD}}_{4\text{MV}}$) for EBT3 films varies between 0.97 and 0.99. While acknowledging that the kVp spectrum is polychromatic compared to the monochromatic X‐ray beams used by Brown and colleagues,^(3)^ our Rx results for beams with effective energies of ∼32 kV and ∼38 kV (70 kVp and 100 kVp) do not agree with their findings. Regarding the significant differences (approximately 20%) in the EBT3 measured responses, it is possible that the EBT3 batches used in this study might have a different (less water equivalent) sensitive emulsion composition with respect to the ones used by Brown et al.^(3)^ The difference in sensitive emulsion composition may also result in a different intrinsic response for the EBT3 films. These differences are particularly critical at low X‐ray energies.

More studies using different batches of EBT3 films and detailed information about the film components (base polyester layer, sensitive emulsion and their physical thicknesses) will help to elucidate the observed variation of EBT3 responses at low X‐ray energies. A recent work by Massillion et al.^(4)^ found that with EBT3 films for a 50 kVp X‐ray beam (HVL=0.77 mm Al), the Rx, defined as ${\text{netOD}}_{50\text{kV}}/{\text{netOD}}_{6\text{MV}}$, is, respectively, 0.90, 0.89, and 0.93 for the red, green, and blue colors at 1 Gy level (75 dpi resolution). These Rx results are significantly higher than the ones observed in the current study for the softest beams investigated (70 kVp, HVL=3.0 mm Al). However, Massillion and colleagues irradiated the EBT3 films using an in‐air setup which reduces the dose contribution from the scatter radiation. The reduced scatter conditions may have an impact in the EBT3 film response. Interestingly, Massillion et al.^(4)^ reported in their study a relatively small (4%) but statistically significant underresponse for 15 MV X‐rays relative to their reference 6 MV (for 1 Gy dose). In order to verify it, we irradiated EBT3 to 1 Gy dose using 6 MV and 18 MV clinical X‐ray beams at our institution. The netOD for the 6 MV and 18 MV irradiations on the dose range 0.5 to 4Gy for all three channels were within 0.4% and 0.8% for 6 and 18 MV relative to ${}^{60}\text{Co}$ beam, contradicting the findings from Massillion et al. Our 6 and 18 MV results are consistent with the findings from a recent study by Casanova Borca et al.^(10)^ They use EBT3 for intensity‐modulated radiation therapy verifications and showed the energy independence of EBT3 films for 6 MV and 15 MV X‐ray beams. Given the observed EBT3 energy response consistency between 6 MV and ${}^{60}\text{Co}$, a negligible change in the observed EBT3 energy dependence may result if 6 MV is used instead of ${}^{60}\text{Co}$ as a reference beam. The film scanning performed at a higher resolution of 150 dpi (0.169 mm) did not show any statistically significant difference in terms of derived netOD compared to the 72 dpi scanning resolution for all of the irradiated films used in this study.

## V. CONCLUSIONS

EBT3 radiochromic films present significant energy response dependence in the orthovoltage X‐ray energy range. An underresponse of more than 20% was observed for beams of 70 kVp (HVL=3.0 mm Al) with respect to ${}^{60}\text{Co}$ clinical beams. A small but still clinically significant lower response was obtained for 300 kVp, (HVL=4.0 mm Cu) beam. Batch consistency for EBT3 films as evaluated in this study for two EBT3 batches showed a small but systematic difference in the relative response for the lowest kVp energies (∼4%); this effect is significantly reduced for higher kVp X‐ray beams. This variability of batch response for EBT3 films must be addressed by a careful calibration of every EBT3 batch. This strategy will provide a solid base for the use of EBT3 in the orthovoltage X‐ray dosimetry domain.

## ACKNOWLEDGMENTS

The authors wish to thank the staff of the Animal Resource Centre, University of Calgary, for their continued support for this research project. We are particularly grateful to Heather Finch and Dr. Mejid Ayroud.

## Supporting information

Supplementary Material
