# Supplementary material for: Energy response of EBT3 radiochromic films: implications for dosimetry in kilovoltage range
Source: J Appl Clin Med Phys. 2014 Jan 6;15(1):331–8. doi: 10.1120/jacmp.v15i1.4439 (PMC5711253; doi:10.1120/jacmp.v15i1.4439)
Supplement: Supplementary file 1 — Supplementary Material [file ACM2-15-331-s001.docx]

**Energy Response of EBT3 Radiochromic Films - Implications for Dosimetry in Kilo Voltage Range**

**J. Eduardo Villarreal-Barajas* and Rao F.H. Khan**

*Department of Oncology and Department of Physics and Astronomy, University of Calgary, Calgary, AB, Canada*

*Department of Medical Physics, Tom Baker Cancer Centre, Calgary, 1331, 29^th^ street NW, Calgary, AB, Canada T2N4N2, Ph:-403-521-3598, Fax:-403-521-3327*

*Eduardo.villrrealbarajas@albertahealthservices.ca*

Running title: EBT3 dosimetry in kV range
